# Bioadhesive microneedle patches for tissue sealing

**DOI:** 10.1002/btm2.10578

**Published:** 2023-08-01

**Authors:** Eden Freundlich, Neta Shimony, Adi Gross, Boaz Mizrahi

**Affiliations:** ^1^ Faculty of Biotechnology and Food Engineering Technion – Israel Institute of Technology Haifa Israel

**Keywords:** bioadhesive, biomaterials, Carbopol, chitosan, microneedle, sealant, tissue regeneration

## Abstract

Sealing of soft tissues prevents leakage of gas and liquid, closes wounds, and promotes healing and is, therefore, of great significance in the clinical and medical fields. Although various formulations have been developed for reliable sealing of soft tissue, tradeoffs between adhesive properties, degradation profile, and tissue toxicity limit their clinical use. Hydrogel‐based adhesives, for example, are highly biocompatible but adhere very weakly to the tissue and degrade quickly, while oxidized cellulose patches are poorly absorbed and may cause healing complications postoperatively. Here, we present a novel strategy for tissue sealing based on bioadhesive microneedle patches that can spontaneously adhere to tissue surface through electrostatic interactions and swell within it. A series of microneedle patches made of pullulan, chitosan, Carbopol, poly (lactic‐co‐glycolic acid), and a Carbopol/chitosan combination were fabricated and characterized for their use in tissue sealing. The effect of microneedle composition on the fabrication process, physical and mechanical properties, in vitro cytotoxicity, and in vivo biocompatibility were examined. The needle structure enables microneedles to strongly fix onto various tissues via physical interlocking, while their adhesive properties improve staying time and sealing capabilities. The microneedle patch comprising Carbopol needles and chitosan as a second pedestal layer presented the best results in terms of sealing and adhesion, a consequence of the needle's swelling and adhesion features combined with the supportive chitosan base layer. Finally, single Carbopol/chitosan patches stopped intense liver bleeding in a rat model significantly quicker and with less blood loss compared with commercial oxidized cellulose patches. These microneedles can be considered a promising cost‐effective platform for adhering and sealing tissues as they can be applied quickly and painlessly, and require less trained medical staff and equipment.

**Translational Impact Statement**

The introduction of novel, reliable, cheap, and safe surgical sealants and adhesives is expected to create lucrative opportunities for the market.

## 1 INTRODUCTION

Surgical sealants play a crucial role in the closing of wounds, prevention of gas and liquid leakage from wounds, and ultimately in promoting healing.^1^ Although various sealants have been developed for tissue sealing, such formulations are still limited by several shortcomings, specifically, the tradeoff between adhesive properties, the product's degradation profile, and tissue toxicity.^2^ As a result of these limitations, surgeons are forced to choose between extremes of adhesion strength, suitable degradation profile, and biocompatibility.^3^ Cyanoacrylate derivatives, for example, adhere very strongly to the tissue, close and seal its surfaces, but are very fragile and are limited to external tissues due to excessive inflammation upon application.^4^ In contrast, fibrin sealants and other hydrogel‐based adhesives generally have excellent biocompatibility but adhere very weakly to tissue and degrade very quickly because of their high water content and weak structural integrity.^5^ Moreover, crosslinked hydrogels, such as polyethylene glycol (PEG) and polysaccharides, have been associated with tissue injuries and pressure build‐up when applied to closed inner cavities, with excess amounts not removed by irrigation.^6^ To overcome these limitations, several sealants with new structures and chemistries have been suggested including complimentary liquid neat (solvent‐free) pre‐polymers,^7^ use of hot‐melt glue gun using star polycaprolactones with low melting points,^8^ a polymer blend of poly(lactic‐co‐glycolic acid) (PLGA) and poly(ethylene glycol) (PEG) that increases wet tissue adherence by incorporating nano‐to‐microscale silica particles,^9^ and laser‐activated sealants,^10^ to name a few. The clinical efficiency of these sealants is, however, still under investigation.

A microneedle (MN) patch is a minimally invasive apparatus composed of micron‐sized needles arranged on a small patch. The concept of MNs was first conceived in 1976 as an attractive alternative for the transdermal delivery of drugs.^11^ Interest in MN technology has been growing steadily in recent decades^12^ and is expected to keep increasing following the publication by the US Food and Drug Administration (FDA) of its official guidance document entitled “Regulatory Considerations for Microneedling Products”, defining MNs as a device,^13^ in November 2020.^14^

**FIGURE 1:**
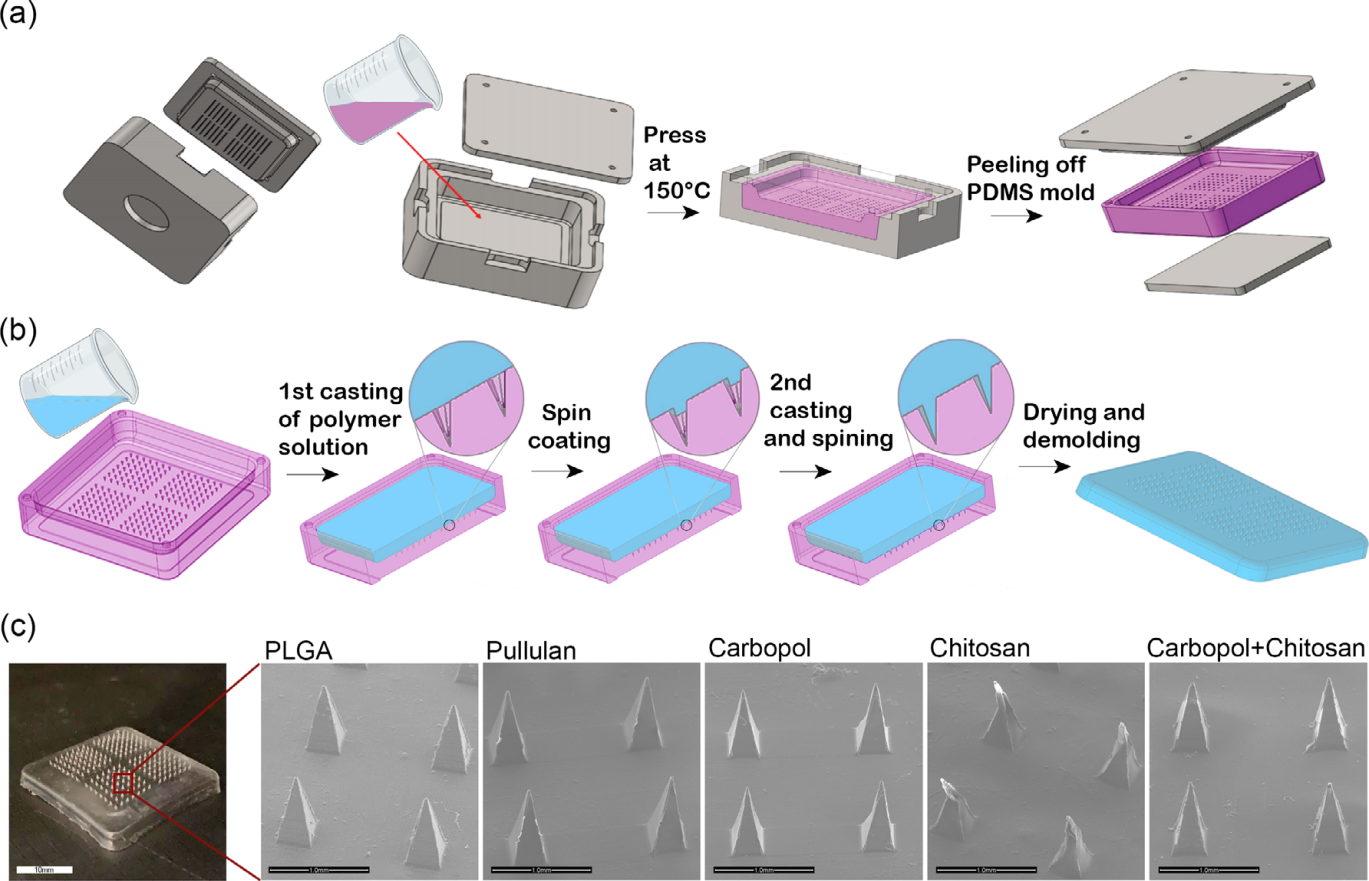
Fabrication of microneedle mold and patches. (a) Fabrication of polydimethylsiloxane (PDMS) mold: PDMS (in pink) was poured into the designated mold (red arrow), sealed with a lid, and cured for 10 min after which the patch was removed from the master mold. (b) The PDMS mold was used to fabricate the various microneedles by dual casting. (c) Image of MN patch (scale bar 10 mm) and SEM images of the various polymeric MNs (scale bar 1 mm).

Here, we propose to rationally design a simple biodegradable adhesive MN patch that would provide a rapid wound/incision closure for internal and external uses. The developed MNs are expected to provide strong and reliable tissue adhesion, be quick to apply, degradable in a relevant time without compromising safety and toxicity. We hypothesize that a sealant in the form of a MN patch using bioadhesive polymers will not only possess the ability to mechanically penetrate and adhere to the tissue, but also the capability to swell within it, further contributing to adhesion. The minimally invasive MNs will mechanically interlock with tissue through swellable MN tips while forming chemical bonds with the tissue thanks to the bioadhesive nature of the polymers. Polymeric MNs will be prepared via the micromolding method^15^ using a self‐made apparatus (Figure 1a). Four mucoadhesive polymers were chosen for the fabrication of the MN patches: (1) Carbopol, a synthetic crosslinked polyacrylic acid with numerous carboxylic acid groups^16^; (2) chitosan, a semi‐synthetic, water‐insoluble (at physiological pH), positively charged copolymer‐derived amino polysaccharide with amino hydroxyl groups^17^; (3) pullulan, a neutral, water‐soluble, linear polysaccharide with a high concentration of hydroxyl groups^18^; and (4) poly (lactic‐co‐glycolic acid) (PLGA), a synthetic non‐swellable, water‐insoluble polyester with negative influence on cell adhesion.^19^, ^20^ The efficacy of the various MNs as surgical sealants was studied in vitro and the most promising formulation was tested in vivo.

## 2 RESULTS

### 2.1 MN fabrication and morphology

Polymeric MNs were fabricated using the micromolding technique. First, a master mold was designed, made of aluminum and Uddeholm Viking using SolidWorks. A polydimethylsiloxane (PDMS) mold of needles with a total height of 900 μm and base edge of 350 μm was fabricated (Figure 1a). Various polymeric solutions were then cast twice in the PDMS mold, spun, and dried for 48 h at room temperature (Figure 1b). MNs were configured in four 7 × 7 two‐dimensional arrays with an array‐to‐array spacing of about 1.5 mm (Figure 1c). Scanning electron microscopy (SEM) confirmed a well‐structured pyramid‐shaped MN configuration with a height and base length of approximately 900 μm and 350 μm, respectively, matching the dimensions of the mold (Figure 1c). Chitosan patches, however, had slightly crooked tips, apparently distorted during removal from the PDMS mold due to the needles' fragile structure.^21^

### 2.2 Compression tests and failure morphology

**FIGURE 2:**
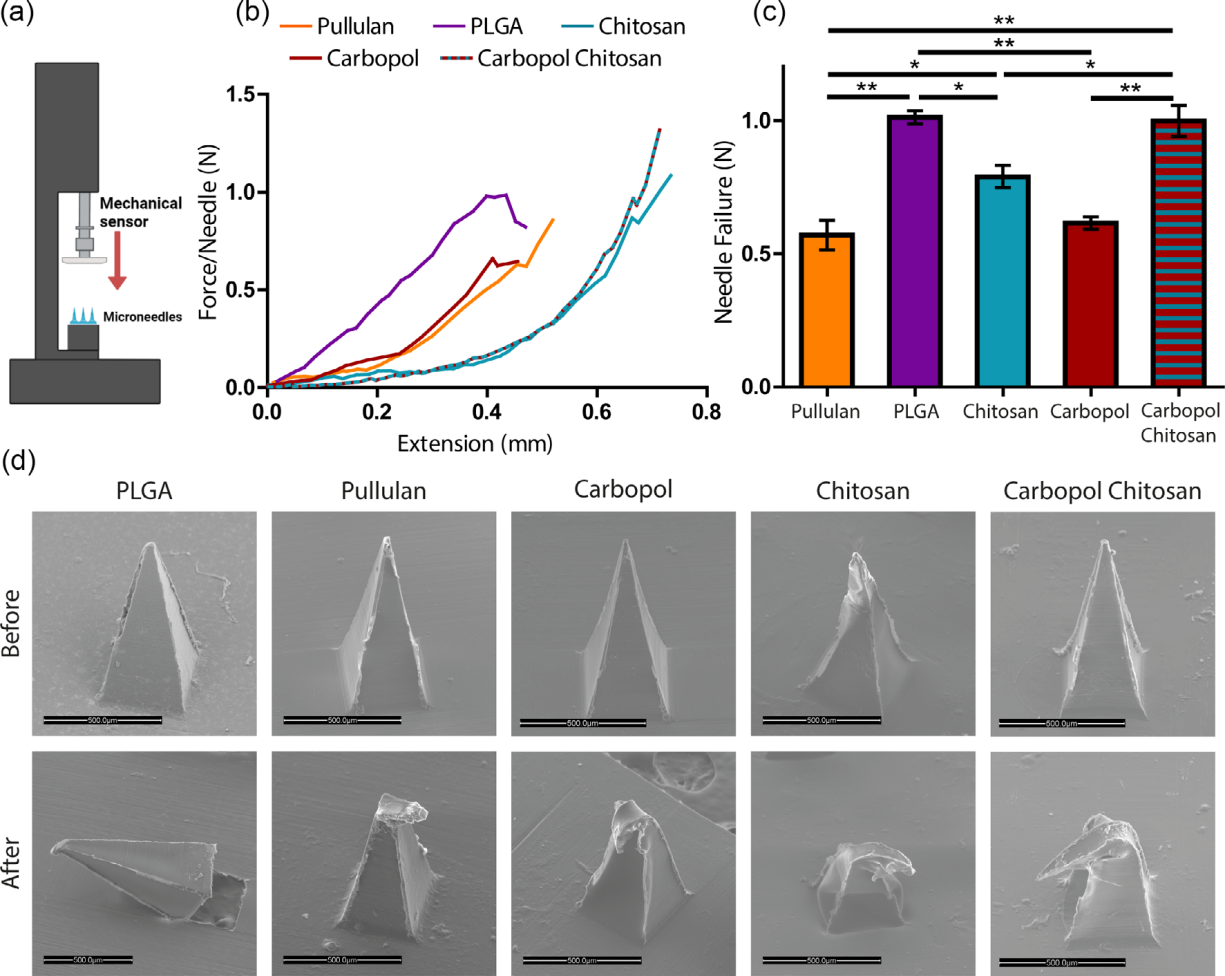
Mechanical properties of the MN patches. (a) Illustration of the texture analyzer set up. (b) Representative force‐extension curves. (c) Needle failure (*n* = 4, *p < 0.05 and **p < 0.005). (d) Needle before and after failure test (scale bar 500 μm).

Insertion force, defined as the force required to puncture the tissue, is an important parameter in the estimation of MN injectability. To determine whether the fabricated patches are strong enough to withstand the insertion force, patches (3 × 3 needles) were placed on the lower plate of a texture analyzer and were subjected to compression by the moving upper plate (Figure 2a). Failure point was defined as the maximum force detected before either sudden decrease or saturation occurred.^22^ The failure force per MN was obtained by dividing the maximum force by the number of MNs compressed until the needle broke (Figure 2b). The average strength of the MNs was determined as the force at which the structure breaks (Figure 2c). Failure forces for the tested patches ranged between 0.57 N/needle (for pullulan) and 1 N/needle for the PLGA and Carbopol/chitosan MNs. SEM analysis was performed to evaluate the effect of compression tests on the morphology and integrity of the needles. Before fracture tests, all needles displayed a complete pyramidal shape integrated into the base substrate. The base and surface of the needles were smooth without the presence of any cracks or fractures, indicating the structural integrity of the patch. After fracture tests, all MNs except for the PLGA showed clear signs of deformation limited to the tips of the needle (Figure 2d). PLGA MNs, on the other hand, broke at the interface between the needles and the base but showed no major deformation or cracks at the tip. The morphological differences between PLGA patches and all other patches can be attributed to the elastic nature of the water‐soluble polymers compared with PLGA,^23^ particularly at the tip areas.^24^

### 2.3 Burst pressure and adhesive strength

**FIGURE 3:**
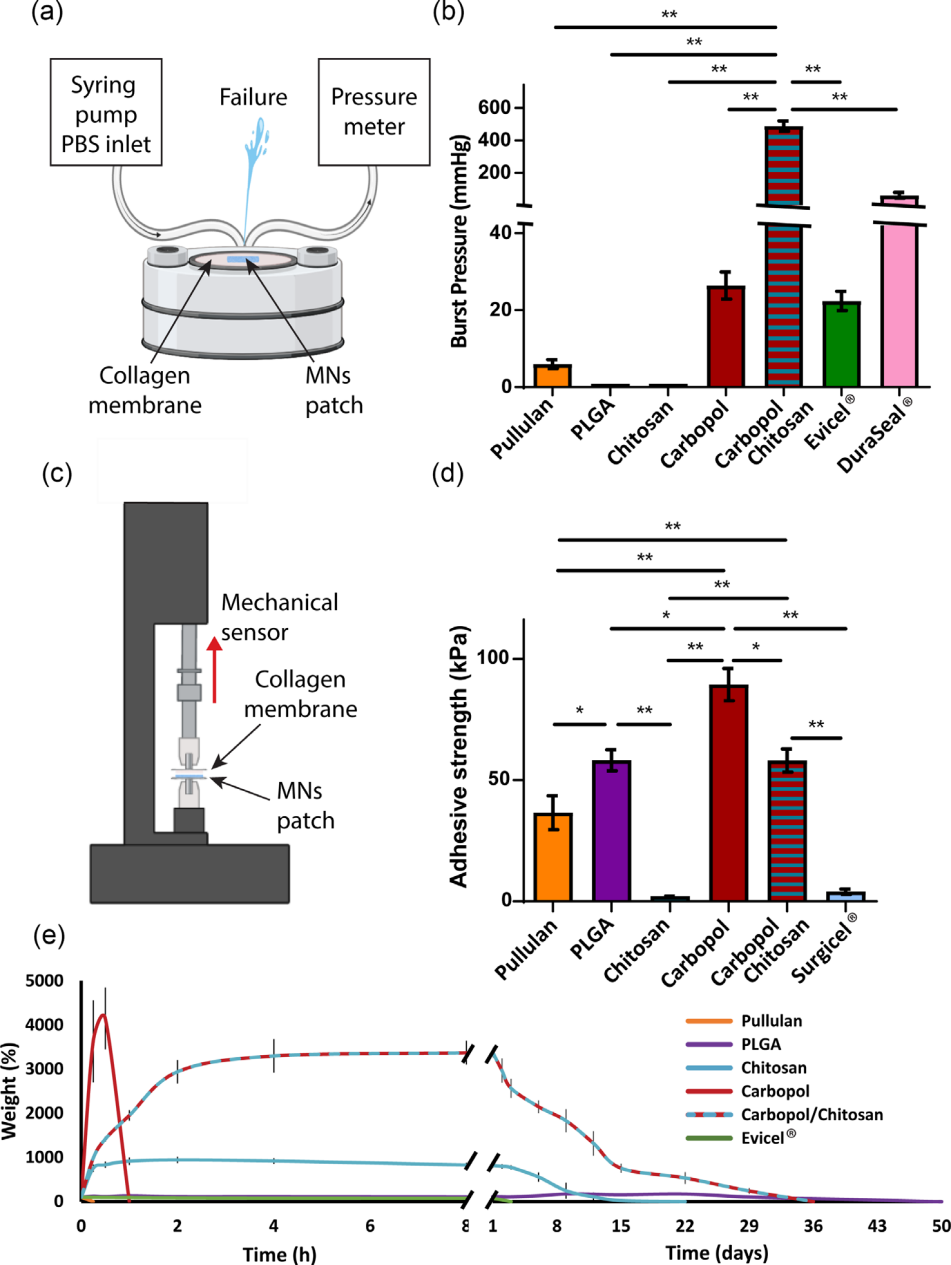
Characterization of the microneedle patches. (a) Schematic illustration of the burst pressure testing system. The membrane with the MN patch is placed in the center of the burst instrument and pressure is created using a syringe pump. Failure occurs when the MN cannot withstand the pressure. (b) Burst pressure results for MNs made of different polymers and for Evicel® and Duraseal®. (c) Schematic illustration of the adhesion testing system (d) Adhesion results for MNs made of different polymers and Surgicel® (e) Swelling and erosion results in PBS at 37°C (*n* = 4, *p < 0.05, **p < 0.001).

To serve as tissue sealants and prevent leakage, MNs must withstand pressure after administration. In this experiment, a burst pressure apparatus was used to test the patches for their ability to seal soft tissue (mimicked by collagen membrane; Figure 3a). The burst pressure strengths of the single‐material MNs were 0 mmHg for PLGA and chitosan, 6 mmHg for Pullulan, and 26 mmHg for pure Carbopol (Figure 3b). Evicel® presented a burst strength of around 22 mmHg and DuraSeal® presented a strength of 100 mmHg (Figure 3b). On the other hand, MNs composed of Carbopol/chitosan exhibited significant higher strength compared with all other MNs tested, with values around 480 mmHg. Since surgical sealants are required to withstand physiological blood pressures ranging from 25 mmHg for capillary bleeding to 120 mmHg for larger blood vessels under normal physiologic conditions,^25^ it is clear that only the Carbopol/chitosan MN patches possess satisfactory mechanical properties that meet clinical requirements. The adhesive strength of the MNs on collagen membrane was further quantified after being stretched at a rate of 30 mm/min. The peak detachment force (kPa) was calculated from the force (N) versus extension (mm) diagram (Figure 3d). The pure Carbopol MNs presented the highest adhesive strength, around 90 kPa, followed by the Carbopol/chitosan and the PLGA MNs, both of which had an adhesive strength of 60 kPa, and the Pullulan MNs with 35 kPa. The pure chitosan MNs, as well as the Surgicel® patches exhibited significantly lower strength, around 3 kPa.

### 2.4 Swelling and erosion

Surgical sealants are designed to prevent leakage of fluids from injured tissue by swelling and filling the irregularly shaped wound, remaining there for several days. While the pullulan MNs dissolved within a few minutes, Carbopol patches swelled by more than 4000% in the first hour and formed a hydrogel‐like substance that eroded within the following hour (Figure 3e). Chitosan MNs also swelled considerably, around 900% within 2 h, followed by a plateau for 24 h and gradual erosion lasting 22 days. The hydrophobic PLGA MNs exhibited minimal swelling, yet lost their initial shape within 7 days and fully degraded within 60 days. Carbopol/chitosan patches showed high swelling degree within 4 h, followed by a plateau for additional 20 h. Then, patches eroded gradually until fully dissolved in 36 days. For comparison, this experiment was also performed on Evicel® (commercial fibrin glue), which did not swell and fully degraded within 3 days.

### 2.5 In vitro cytotoxicity

**FIGURE 4:**
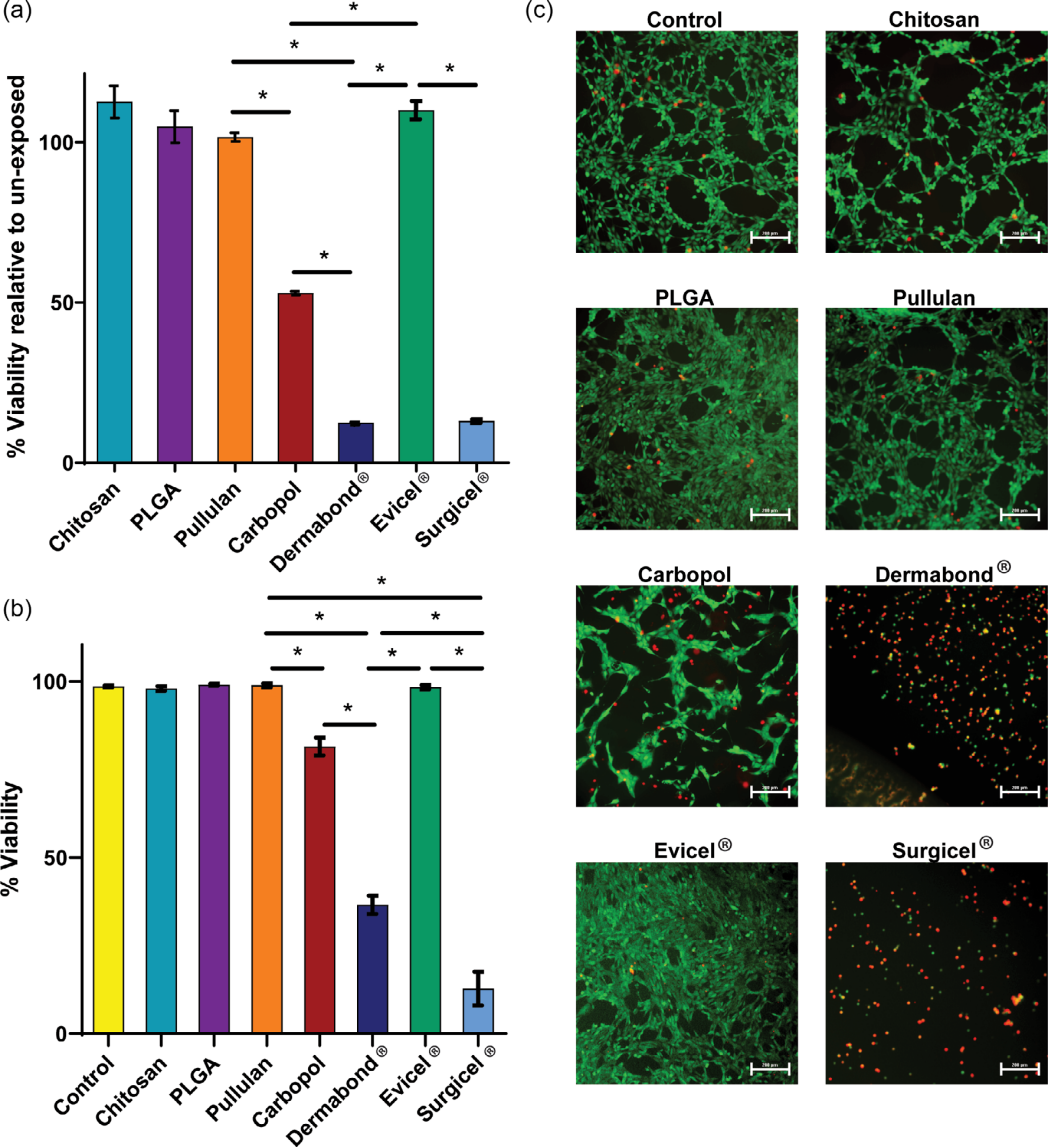
Cytotoxicity of the MN patches (*n* = 4, *p < 0.005). NIH 3T3 cell viability compared with unexposed cells, evaluated 48 h after exposure to the MN patches or Dermabond® by MTS assay (a) and live/dead assay (b). (c) Fluorescence microscopy (live—green, dead—red) of cell response to MN patches using live/dead assay (scale bar 200 μm).

Cytotoxicity of the different MN patches was evaluated in vitro using NIH 3T3 fibroblast cell lines and was compared with that of Dermabond®, Evicel®, and Surgicel®. Cells exposed to chitosan, PLGA, Pullulan, and Evicel® retained viability of around 100% compared with unexposed cells. Exposure to Carbopol MNs resulted in 50% cell viability while exposure to Dermabond® and Surgicel® led to 20% and 10% viability, respectively (Figure 4a). The live/dead assay^26^ (Abcam, Cambridge, England) mirrored the MTS assay results: the majority of cells exposed to chitosan, PLGA, Pullulan, and Evicel® remained alive compared with the control cells, which were not exposed to the MNs. Cells exposed to Carbopol, however, presented viability of around 80% in the live/dead assay, higher than the outcome of the MTS assay. In contrast, almost no living cells were observed in the Dermabond® and Surgicel® groups (Figure 4b,c).

### 2.6 In vivo efficacy assessment

**FIGURE 5:**
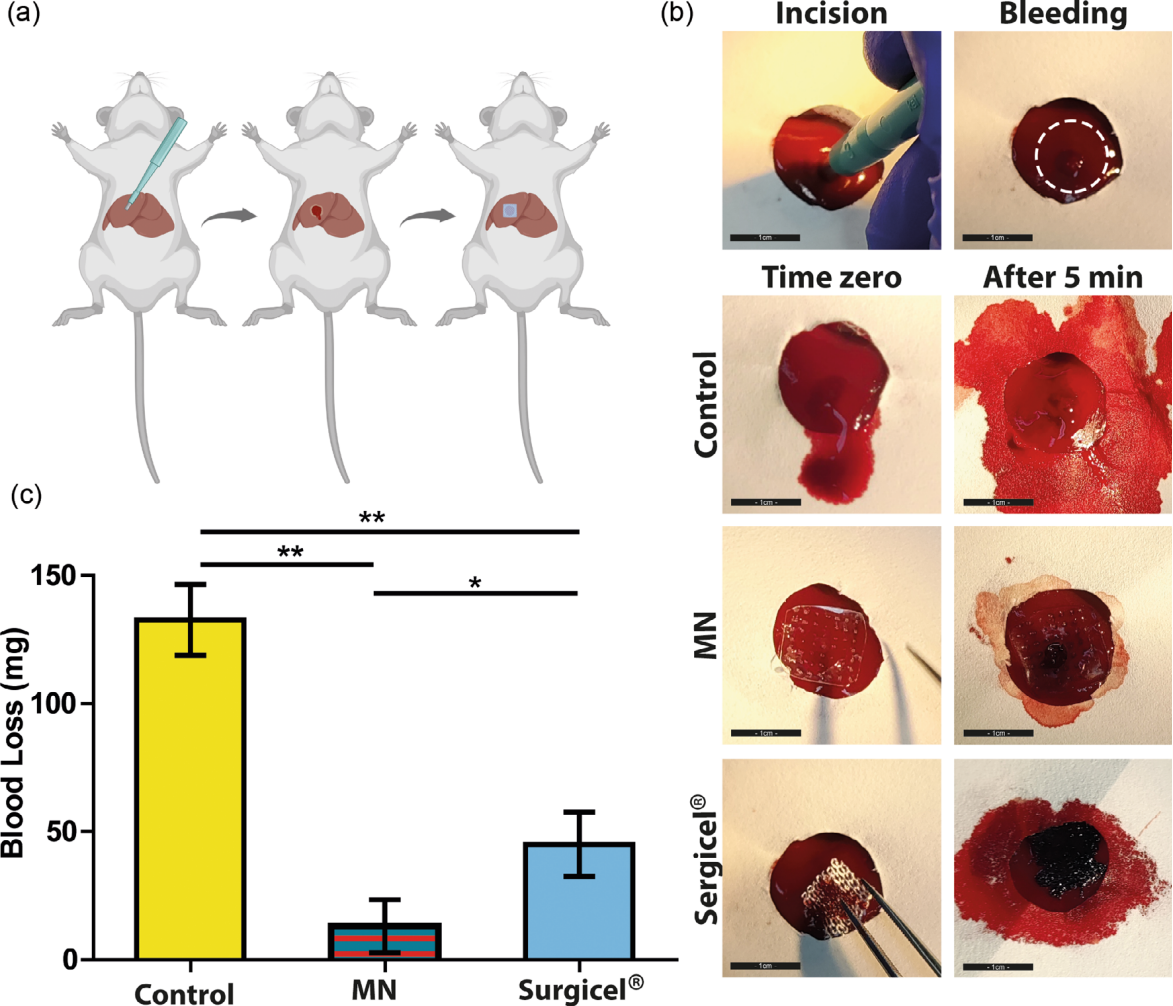
(a) Schematic illustration of the incision procedure and the MN patch treatment on rat liver. (b) Incision and treatment of rat liver bleeding model without any treatment (control), with MN patch, and with Surgicel®, respectively (scale bar 1 cm). (c) Blood loss for the three treatment groups (*n* = 4, *p < 0.05, **p < 0.01).

MN patches made of chitosan and Carbopol were chosen for this assay since this material combination yielded the most promising results on the burst pressure and swelling and erosion tests. A rat liver bleeding model was used to study the potential of the adhesive MNs as surgical sealants. The model was created by making an incision in a rat liver lobe, after which the lobe began bleeding, and a MN patch was applied directly on the incision area (Figure 5a,b). The highest blood loss, 132.7 ± 13.8 mg, was observed for the non‐treated livers followed by livers treated with Surgicel®, which lost 45.1 ± 12.6 mg, and livers treated with the double‐layered MN (Carbopol/chitosan), which lost 13.1 ± 10.4 mg of blood. Thus, in both treated groups bleeding was significantly reduced. The results also elucidate the superiority of our MN patch over the oxidized cellulose, as bleeding was restricted to the first 5 s, and blood loss was significantly lower than using the commercial hemostat (*p* < 0.05).

## 3 DISCUSSION

In this study, we suggest a different approach for tissue sealing that combines the advantages of invasive techniques such as suture and staples (i.e., strong and reliable) with those of glues (i.e., amenable to quick application, simple application, and biodegradability). Our structure motif is based on the fabrication of a MN device, that is, a minimally invasive apparatus composed of micron‐sized needles arranged on a small patch that forms strong chemical, physical, and mechanical interlocking with the tissue. Moreover, we chose to focus on both the composition and the structure of the sealant. The MNs we tested presented a very wide range of mechanical properties, due to the various properties of the biomaterials used in their fabrication. The Carbopol/chitosan MNs offered another level of sophistication in which one material is used for fixation and adhesion while the other provides support, long‐term adhesion, and degradation control.

The two Carbopol‐containing MNs exhibited enhanced sealing and adhesive strength. Carbopol is a bio‐adhesive, anionic, and cross‐linked polymer of acrylic acid with a high molecular weight. Carbopol's adhesion mechanism can be divided into two steps: first, the “contact stage,” characterized by the polymer spreading and swelling on the surface of the tissue; and second, the “consolidation stage,” in which Carbopol adhesion is activated by the presence of moisture.^27^, ^28^ This activation is attributed to hydrogen bond building groups (—OH, —COOH), to the high molecular weight of Carbopol, and to its chain flexibility.^29^ In addition, at physiological pH, Carbopol is negatively charged (pKa of around 5), which further contributes to cell adhesion via electrostatic interactions.^30^, ^31^ Nevertheless, the pure Carbopol patches presented the highest adhesive strength (90 kPa, Figure 3d) and the second highest average burst pressure values (~30 mmHg). Carbopol patches also swelled excessively and eroded within an hour, making them clinically irrelevant. The patch fabricated from Carbopol and chitosan, that is, from Carbopol needles and a pedestal layer made of chitosan, overall presented the most promising properties of the various patches (Table S1). MNs made of this combination exhibited the highest needle failure point (1 N/needle) without compromising elasticity required to maintain the integrity of the needles during penetration. The Carbopol/chitosan MNs also had the highest burst strength, 10‐fold higher than that of the pure Carbopol MN. The excellent performance of the double‐layered MNs can thus be attributed to the role of each layer: the stiff Carbopol needles allow easy and comfortable insertion into wet tissue (fixing the patch via physical and electrostatic interlocking) while the elastic chitosan layer absorbs the interfacial water and becomes a soft hydrogel. The elasticity of chitosan patches and their ability to quickly absorbed water was evident in this study and by others.^32^ Thus, a strong reinforcing base layer is crucial for appropriate sealing, as was evident from the burst pressure and the erosion studies. This was also evident by the pure chitosan patch that barely adhered to the collagen membrane and, therefore, failed to withstand any fluid pressure. The fact that these patches degraded within 3 weeks under physiological conditions demonstrated the notion that only by combining the two polymers, a long‐lasting adhesive MN patch can be obtained.

Several surgical sealants are currently available for reconnecting or sealing of tissues during surgical intervention.^33^, ^34^ Oxidized cellulose, such as Surgicel®, has traditionally been used as a hemostatic agent thanks to its similarity to the extracellular matrix, porous structure, and clinical performance.^35^ However, oxidized celluloses have some inherent drawbacks that make it far from ideal. First, the hemostatic property of oxidized celluloses is local and relatively weak, and a single patch cannot guarantee *favorable clinical outcomes*.^36^ In addition, oxidized celluloses are contraindicated for treatment of several sensitive tissues such as nervous and cardiac systems, due to the high carboxylic content of the entire matrix.^35^ Finally, oxidized celluloses are ineffective in patients with blood coagulation disorders since they only provide a strong matrix for platelet adhesion but have no intrinsic hemostatic effect.^37^ Other biomaterials such as cyanoacrylates are very strong but also very toxic and are restricted to external use.^38^ This toxicity was demonstrated in our in vitro experiment in which all cells around the cyanoacrylate did not survive. Finally, fibrin sealants, although safe and approved for internal use by the FDA,^39^ suffer from weak structure integrity and adhesion and quickly clear from the site of injection,^7^ as was demonstrated in our in vitro swelling and erosion study. Consequently, new methods for the control of bleeding and tissue sealing, such as the one presented in this study, are much needed.

## 4 CONCLUSION

We developed MN patches for tissue sealing from biofriendly, adhesive materials. Patches made of Carbopol/chitosan showed the most promising results in terms of mechanical properties, swelling and erosion profiles, and sealing strength. The combination of the two different layers also created long‐lasting patch with excellent adhesion strength. These properties were demonstrated in a liver bleeding model. Finally, these MNs do not require expensive fabrication processes and can, therefore, be viewed as a promising cost‐effective platform for adhering and sealing organs. In appropriate circumstances, such a technique can offer attractive alternatives to sutures, staples, and glues since they can be applied quicker, causes less pain, and require less trained medical staff and equipment.

## 5 EXPERIMENTAL SECTION

### 5.1 Materials

Medium molecular weight chitosan, was purchased from Sigma Aldrich (USA), pullulan was purchased from Alfa Aesar (USA), and Carbopol (Carbomer 941, NF) was purchased from Spectrum (USA). Poly(lactic‐co‐glycolic acid), with lactide and glycolide at a 50/50 molar ratio (Resomer® RG 503 H), was purchased from Evonik (Germany), acetic acid glacial was purchased from Carlo Erba (Italy), and SYLGARD™ 184 silicone elastomer kits were purchased from Dow Corning (USA). Acetonitrile was purchased from JT Baker (USA), and 2‐octyl‐CA (Dermabond®), Evicel®, and Surgicel® were purchased from Ethicon Inc. (USA). Duraseal® was purchased from Integra LifeSciences Corporation (France). Live/dead cell viability assay kits were purchased from Abcam (England), and CellTiter 96 Aqueous One Solution Cell Proliferation Assay (MTS) kits were purchased from Promega (USA). Penicillin–streptomycin, fetal bovine serum, and l‐glutamine were purchased from IM Beit HaEmek (Israel). Pre‐hydrated collagen membranes were purchased from Vista International Packaging (USA), POLYDINE® was purchased from Dr. Fisher (Israel), and buprenorphine (1.3 mg/mL) was purchased from LogiPharm Ltd. (Israel).

### 5.2 Design and fabrication of the master mold

A master mold made of aluminum and Uddeholm Viking, an oil‐air‐vacuum‐hardening steel, was designed by SolidWorks and manufactured by Amir Perle Engineering Ltd. (Israel). The design, which comprises an array of needles with a total height of 900 μm and base edge of 350 μm, is based on the notion that pyramidal needles have improved penetration capabilities compared with conical needles.^40^ Molds were then manufactured from PDMS (10:1 w/w elastomer and curing agent ratio) using the master mold (see Figure 1a). PDMS solution was poured into the bottom part of the master mold, and the needle‐studded lid was lowered over the bottom part of the mold, displacing excess PDMS solution to form the designated holes. The filled master mold was then placed on a bench top standard heated press (Carver Inc., USA; Model 3856) and pressed at 150°C for 10 min. Finally, the PDMS mold was gently peeled off the master mold.

### 5.3 Fabrication of MN patches

Various polymeric MNs were prepared using a micromolding technique.^41^ Polymeric solutions were prepared as follows: Carbopol 2% (w/v) in double distilled water (DDW); Pullulan 5% (w/v) in DDW, PLGA 5% (w/v) in acetonitrile; and chitosan 2% (w/v) in 1% (v/v) acetic acid (1% acetic acid in DDW). Then, 500 μL of each of the above‐mentioned polymeric solutions were placed in the PDMS mold and pulled into the cavities using a spin coating device (Laurell Technologies, USA; Model WS‐650MZ‐23NPPB) at 10,000 rpm for 10 min. The mold was then topped off with polymeric solution (500 μL) followed by a second centrifugation at 10,000 rpm for 10 min. After the centrifugation process, about 5 mL of the polymeric solution was used to fill the mold. Molds were allowed to dry for 48 h at room temperature before MNs were carefully peeled off the mold. The only exception to this procedure was the fabrication of the Carbopol‐chitosan MNs where an additional step was carried out: After complete drying of the Carbopol MNs, 5 mL of chitosan solution was poured into the mold (creating the pedestal layer). The surface morphology of all polymeric MNs was investigated using a wide‐field scanning electron microscope (the Netherlands; Model Quanta 200 FEI ESEM), with an accelerating voltage of 20 kV. Samples were not coated prior to imaging.

### 5.4 Compression tests and failure morphology

Insertion force, defined as the force required to puncture the tissue, is an important parameter in the evaluation of the MNs. Needle strength, that is, the force required to cause needle failure, was evaluated by applying an axial compression load using a TA1 texture analyzer (Lloyd, AMETEC, USA) equipped with a 50 N load cell. MN patches (3 × 3 needles) were placed on the lower plate of the texture analyzer using double‐sided tape and were subjected to compression by the moving upper plate. The pre‐test and post‐test speeds were 2 mm/min and 1 mm/min, respectively, and the trigger force was set at 0.1 N. Data were collected until the needles broke and were displayed as force per needle versus extension. The post‐test morphology and integrity of needles were determined by SEM (the Netherlands; Model Quanta 200 FEI ESEM).

### 5.5 Burst pressure and adhesive strength

The objective of this test was to quantify the maximum pressure withstood by the MNs at the tissue leakage point. Burst strength of the patches was tested on soft tissue using the standard test method for surgical sealants (ASTM F2392‐04^42^). In brief, dried collagen membranes (Vista International Packaging, USA) were punctured using a 3 mm puncher and sealed with the tested MN patches. Membranes were then clamped down and pressure was applied by using a Master Dual Pump (Braintree Scientific Inc., USA) to infuse PBS buffer (pH 7.4) into the burst system at a constant rate of 1 mL/min. Pressure in the membrane was monitored throughout the experiment using a pressure gauge (Lutron, USA; Model PS‐9302). Burst pressure was defined as the pressure at which the sealant failed and the membrane started leaking. In all of the burst pressure tests, failure took place around the puncture site, confirming that the internal burst pressure of the collagen membranes was higher than that of the tested materials. The tissue adhesion strength of the MNs was investigated using an EZ50 tensile testing machine (Lloyd Instruments, Denmark) and 1 × 1 cm collagen membrane (Vista International Packaging, USA) immersed in PBS (pH 7.4) for 5 min before the test. Each MN was glued to the stationary crimp using ethyl cyanoacrylate adhesive (3 M, MN, USA). Then, MN were inserted into the wet collagen membranes and a contact force of 150 g was maintained for 4 h, after which the probe was withdrawn from the membrane at a rate of 30 mm/min and the adhesive strength (kPa) was recorded. Adhesive strength was determined as the pressure at which the maximal point, before a sharp decrease, attributed to the disconnection between the tablet and the membrane, was observed. Results are presented as averages ± SD and compared with Surgicel® (*n* = 5).

### 5.6 Swelling and erosion

The swelling and erosion of the different MN patches were measured by incubating each patch in 30 mL of PBS (pH 7.4) at 37°C with constant shaking (60 rpm). MN patches were weighed at predetermined times after careful blotting with filter paper and returned immediately to the incubator to continue the experiment. The total percent of material weight was calculated by dividing the wet weight (Ww) of the swollen sample by its initial dry weight prior to incubation (Wd = 40 mg at *t* = 0). The results are reported as means ± SD (*n* = 4).

### 5.7 In vitro cytotoxicity

NIH 3T3 fibroblast cells were grown at 37°C in Dulbecco's modified Eagle's medium (DMEM) supplemented with 10% fetal bovine serum (Gibco‐Invitrogen Corp., USA). Cultures were maintained in a 95% air/5% carbon dioxide atmosphere, at 95% relative humidity. MN patches were sterilized by exposure to UV light for 15 min. The cytotoxicity of the MN patches was studied by exposing cell lines to 3 × 3 tested needles. Cytotoxicity was assessed 48 h after cells were added, using MTS kits^43^ (CellTiter 96 Aqueous kit, Promega, USA) and live/dead fluorescence viability kits^26^ (Abcam, England). The results are reported as means ± SD (*n* = 4).

### 5.8 Acute in vivo sealing model

All animal experiments were performed in strict accordance with the Guide for the Care and Use of Laboratory Animals and were approved by the Council for Animal Experiments, Israel Ministry of Health (Animal Ethics Approval No. IL‐151‐10‐2022). Eighteen female Sprague Dawley rats weighing 190–220 g were purchased from Envigo, Israel. All animals were maintained in sterilized cages on a 12‐h light/12‐h dark cycle with food and water provided. Rats were anesthetized using isoflurane and injected with 0.05 mL of a 1.3 mg/mL long‐acting buprenorphine solution. Rat abdomens were shaved and sterilized with iodine solution (Polydine, Dr. Fischer, Israel) followed by 70% ethanol. A liver bleeding model was then created^44^ by exposing the rat liver, placing filter paper on top, and making a 2 mm deep incision at the center of the lobe using a 3 mm puncher. A two‐layered MN patch (Carbopol/chitosan) was gently pressed over the bleeding area for 5 seconds. Blood loss was measured by weighing the filter paper around the bleeding area before and at 5‐min post‐procedure. For comparison, one group of rats was treated with Surgicel® and a third group remained untreated. The results are reported as means ± SD (*n* = 4).

### 5.9 Statistical analysis

All data were reported as means ±SEM from separate experiments. Statistical analyses were performed by *t*‐test or one‐way ANOVA using the GraphPad Prism (GraphPad Software, USA); *p* values <0.05 were considered statistically significant (*n* = 4).

## AUTHOR CONTRIBUTIONS

**Eden Freundlich:** Data curation (equal); formal analysis (equal); investigation (equal); methodology (equal); writing – original draft (equal). **Neta Shimony:** Data curation (equal); formal analysis (equal); investigation (equal); methodology (equal); writing – review and editing (equal). **Adi Gross:** Funding acquisition (equal); project administration (equal); supervision (equal); writing – original draft (equal). **Boaz Mizrahi:** Conceptualization (equal); funding acquisition (equal); project administration (equal); supervision (equal); writing – review and editing (equal).

## CONFLICT OF INTEREST

The authors have no conflicts of interest to declare.

### PEER REVIEW

The peer review history for this article is available at https://www.webofscience.com/api/gateway/wos/peer-review/10.1002/btm2.10578.

## Supporting information


**Table S1:** Summary of mechanical properties of the tested MNs.

## Data Availability

Data sharing is not applicable to this article as no new data were created or analyzed in this study.
