# Supplementary material for: Bioadhesive microneedle patches for tissue sealing
Source: Bioeng Transl Med. 2023 Aug 1;9(3):e10578. doi: 10.1002/btm2.10578 (PMC11135150; doi:10.1002/btm2.10578)
Supplement: Supplementary file 1 — Table S1: Summary of mechanical properties of the tested MNs. [file BTM2-9-e10578-s001.docx]

Supporting Information

**Bioadhesive Microneedle Patches for Tissue Sealing**

Eden Freundlich*, Neta Shimony*, Adi Gross, Boaz Mizrahi^#^

Faculty of Biotechnology and Food Engineering, Technion – Israel Institute of Technology, Technion City, Haifa 3200003, Israel.

^*^ Equal contribution
^#^ Corresponding author, E-mail: bmizrahi@technion.ac.il

**Table S1**: Summary of mechanical properties of the tested MNs.

|  | Pullulan | PLGA | Chitosan | Carbopol | Carbopol /Chitosan |
| --- | --- | --- | --- | --- | --- |
| Compression force  (N/needle) | 0.6 | 1 | 0.85 | 0.7 | 1 |
| Adhesion test (KPa) | 35 | 60 | 3 | 90 | 60 |
| Burst pressure (mmHg) | 3 | 0 | 0 | 27 | 440 |
| Erosion (days) | 0.0007 | 50 | 22 | 0.04 | 36 |
| Swelling (%) | 0 | 160 | 900 | 4000 | 3000 |
